# Mechanisms of Resistance in Gastroenteropancreatic Neuroendocrine Tumors

**DOI:** 10.3390/cancers14246114

**Published:** 2022-12-12

**Authors:** Chanjuan Shi, Michael A. Morse

**Affiliations:** 1 Department of Pathology, Duke University Medical Center, Durham, NC 27710, USA; 2 Department of Medicine, Duke University Medical Center, Durham, NC 27710, USA

**Keywords:** somatostatin analog, tyrosine kinase inhibitor, mTOR inhibitor, peptide receptor radiotherapy

## Abstract

**Simple Summary:**

Advanced neuroendocrine tumors originating in the intestinal tract and pancreas are treated with biologics that activate somatostatin receptors (lanreotide and octreotide), small molecule drugs that target the mTOR (everolimus) and VEGF and other signaling pathways (sunitinib), chemotherapies (temozolomide, capecitabine, fluorouracil, platinums), and receptor-targeted radionuclides (Lu177-DOTATATE). These treatments eventually fail to control tumor progression, but the mechanisms for therapeutic resistance are poorly understood. We will review preclinical and early clinical studies that provide insight into potential etiologies and new therapies and combinations that may address this resistance.

**Abstract:**

Gastroenteropancreatic neuroendocrine tumors (GEP-NETs), although curable when localized, frequently metastasize and require management with systemic therapies, including somatostatin analogues, peptide receptor radiotherapy, small-molecule targeted therapies, and chemotherapy. Although effective for disease control, these therapies eventually fail as a result of primary or secondary resistance. For small-molecule targeted therapies, the feedback activation of the targeted signaling pathways and activation of alternative pathways are prominent mechanisms, whereas the acquisition of additional genetic alterations only rarely occurs. For somatostatin receptor (SSTR)-targeted therapy, the heterogeneity of tumor SSTR expression and dedifferentiation with a downregulated expression of SSTR likely predominate. Hypoxia in the tumor microenvironment and stromal constituents contribute to resistance to all modalities. Current studies on mechanisms underlying therapeutic resistance and options for management in human GEP-NETs are scant; however, preclinical and early-phase human studies have suggested that combination therapy targeting multiple pathways or novel tyrosine kinase inhibitors with broader kinase inhibition may be promising.

## 1. Introduction

Gastroenteropancreatic neuroendocrine tumors (GEP-NETs) are rare, but the incidence and prevalence are rising such that, as a group, they are the second most prevalent gastrointestinal cancer after colorectal cancer. Many patients with GEP-NET have metastatic disease at initial presentation [1]. Clinical presentations of GEP-NETs are variable, ranging from an indolent to a very aggressive clinical course, and may be dominated by the morbidity associated with hormonal secretion by functional tumors. While debulking surgery and locoregional therapies are an option for some, in the majority, progressive disease eventually requires systemic therapy to control hormonal-driven syndromes for functional GEP-NETs and to suppress the tumor growth of both functional and nonfunctional GEP-NETs.

Current systemic treatment options for advanced GEP-NETs may be grouped into three main categories: alkylating agent-based chemotherapy, molecularly targeted agents, and somatostatin receptor (SSTR)-targeted therapies (as reviewed in [2].) Molecularly targeted agents include mTOR inhibitors and multi-kinase inhibitors. The mTOR inhibitor everolimus has been approved by FDA for treating unresectable/metastatic gastrointestinal, pancreatic NETs (Pan-NETs), and pulmonary NETs, and the multi-kinase inhibitor sunitinib has been approved for PanNETs. The multikinase inhibitor surufatinib, although meeting its endpoints for progression-free survival compared with placebo in pancreatic and extrapancreatic NETs, has not been FDA-approved. SSTR-targeted therapies include somatostatin analogs (SSAs) and peptide receptor radiotherapy (PRRT) with radiolabeled SSAs. Progression-free survival compared with placebo or standard therapies has been demonstrated for these therapies [2]; however, the eventual development of therapeutic resistance remains one of the major challenges, and most patients eventually succumb to the disease. This review is focused on mechanisms underlying the resistance to molecular- and SSTR-targeted therapies in GEP-NETs.

## 2. Materials and Methods

Articles available through a search of Pubmed or reported in abstract form were the basis for this review.

## 3. Resistance to Molecularly Targeted Therapies

Patients who have primary resistance to a molecularly targeted therapy do not respond to the initial therapy; a lack of targeting molecules and presence of activated alternative pathways are the main mechanisms for primary resistance. Patients with secondary/acquired resistance demonstrate an initial treatment response followed by relapse or progression. Multiple mechanisms underlying secondary resistance to targeted therapies in GEP-NET have been proposed.

### 3.1. Molecular Mechanisms of Resistance to Everolimus

**Figure 1:**
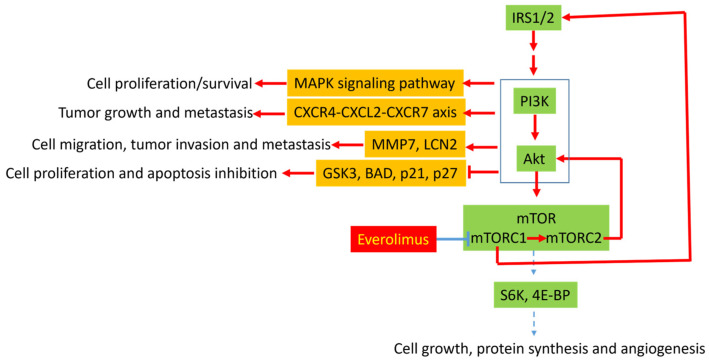
Potential mechanisms underlying everolimus resistance in gastroenteropancreatic neuroendocrine tumors. Inhibition of mTORC1 by everolimus may leads to activation of mTORC2 and elevate insulin receptor substance 1 and 2 (IRS1/2) via disrupting mTORC1-mediated negative feedback. IRS1/2 leads to activation of PI3K/Akt signaling pathway. In addition, mTORC2 also activates Akt by phosphorylation. Activation of PI3K/Akt leads to cell proliferation, tumor growth, apoptosis inhibition, tumor cell migration, tumor invasion, and metastasis through (1) activation of MAPK signaling pathways, (2) activation of CXCR4-CXCL2-CXCR7 axis, (3) overexpression of MMP7 and LCN2, and (4) inhibition of GSK3, BAD, p21, and p27.

PI3K/Akt/mTOR signaling is frequently dysregulated in GEP-NETs [3]. Next generation sequencing studies have revealed that approximately 14–29% of sporadic PanNETs harbor mutations in the mTOR pathway genes [4,5], and immunohistochemistry has demonstrated positive phosphorylated-Akt (p-Akt) in most GEP-NETs, suggestive of activated PI3K/Akt/mTOR signaling in these tumors [6]. Potential mechanisms leading to the activation of the pathway include mutations in the signaling pathway genes and dysfunctional tyrosine kinase receptors involving the signaling pathway [7]. p-Akt activates a number of downstream substrates, including mTOR, which has two different complexes: mTORC1 and mTORC2. mTORC1 is rapamycin-sensitive and activates S6K and 4E-BP, which promotes the translation of proteins involved in cell cycle progression, including cyclin D1, c-MYC, and hypoxia-induced factor 1α (HIF-1α). In contrast, mTORC2 is rapamycin-insensitive and regulates the activity of Akt via a feedback circuit. Other downstream substrates of Akt include glycogen synthase kinase-3 (GSK3), BCL2-associated agonist of cell death (BAD), p21, and p27, which are involved in regulating cell proliferation and apoptosis (Figure 1).

Everolimus, an mTORC1 inhibitor, has demonstrated efficacy in prolonging progression-free survival in PanNETs [8] and GI tract and pulmonary NETs [9]; however, progression-free survival (PFS) ranges from 10.8 to 14.8 months and 9.2–13.3 months, respectively, suggesting the development of acquired resistance. Possible molecular mechanisms leading to everolimus resistance include the mTORC2-dependent and independent activation of Akt (Figure 1). The inhibition of mTORC1 by everolimus increases Akt phosphorylation by mTORC2 [10], which leads to GSK3 inhibition. In addition, mTORC1 inhibition disrupts its negative feedback function by decreasing the proteasomal degradation of insulin receptor substrate (IRS) 1 and 2, thereby leading to the activation of PI3K/Akt. A recent study using two everolimus-resistant PanNET cell lines, however, demonstrated an over-activation of GSK3 and decreased IRS 1evels [11]. Nevertheless, in 50% of patients with solid tumor treated with everolimus, an overexpression of Akt phosphorylation was observed [12]. However, its correlation with everolimus resistance was not explored in the study. A recent study using RNA sequencing and immunohistochemistry revealed an overexpression of C-type lectin domain family 3 member A (CLEC3A), matrix metallopeptidase 7 (MMP7) and lipocalin 2 (LCN2) in recurrent PanNETs compared to non-recurrent tumor [13]. CLEC3A and other molecules activate the PI3K/Akt signaling pathway, leading to an overexpression of MMP7 and LCN2 via an mTOR-independent pathway. Both MMP7 and LCN2 are critical for cell migration, tumor invasion, and metastasis [13]. Therefore, it is reasonable to hypothesize that the PI3K/Akt-mediated overexpression of MMP7 and LCN2 may be a potential mechanism for everolimus resistance in human NETs, and the simultaneous inhibition of both PI3K/Akt and mTOR may overcome the resistance. The dual inhibition of PI3K and mTOR was shown to decrease PanNET metastatic progression in a pancreatic-NET cell line (BON) model [14], further suggesting that the concurrent inhibition of PI3K/Akt and mTOR may reduce everolimus resistance. For clinical use of combination therapy, the toxicities of PI3K inhibition (rash, diarrhea, and hyperglycemia) may be potential challenges; however, a phase Ib study that included a cohort of PanNET patients treated with the PI3Kalpha inhibitor alpelisib and everolimus demonstrated that the combination could be given with dose reductions and that the sixteen-week progression-free survival rate was 35.3% in previously treated PanNET patients and 30.0% in a mixed cohort with prior mTOR inhibitor use [15]. Finally, other approaches to targeting downstream of the PI3K/Akt/mTOR signaling have been attempted in combination with everolimus. LY2584702, an ATP-competitive inhibitor against the downstream p70 S6 kinase, was combined with everolimus in a phase Ib study of patients with advanced solid tumors, but only yielded a stable disease as the best response [16].

The activation of the mitogen-activated protein kinase (MAPK) pathway may be another mechanism for everolimus resistance. Extensive cross-talk is present between the PI3K/Akt and the MAPK signaling pathways, and several key feedback loops between the two signaling cascades have been proposed [17]. Because of the cross-talk, the inhibition of one pathway may lead to the activation of the other. The inhibition of mTORC1 was shown to activate the MAPK pathway through a PI3K-dependent feedback loop in human cancer [18], and rapalog resistance was associated with extracellular signal-regulated kinase 2 (ERK2) upregulation in a human NET cell line [19]. The addition of a MAPK/ERK kinase (MEK) inhibitor to a dual PI3K-mTOR inhibitor significantly enhanced the inhibition of tumor growth by a dual PI3K-mTOR inhibitor alone in human neuroendocrine cell lines [19]. Clinical trials have attempted to combine MAPK pathway inhibition with everolimus, but toxicity has been a challenge. In a phase Ib study of the oral MEK inhibitor trametinib in combination with everolimus in patients with advanced solid tumors (including neuro-endocrine tumors), a phase II dose could not be established due to high rates of mucosal inflammation/stomatitis, fatigue, and diarrhea [20]. A study of the EGFR TKI erlotinib plus everolimus in well- to moderately differentiated neuroendocrine tumors (NCT00843531) was terminated due to poor accrual.

Other receptors that signal through the MAPK and PI3K pathways, including IGF-1R, also play a role in NET growth such that targeting IGF-1R induces the apoptosis of NET cells [21]. Preclinical studies reporting that the inhibition of IGF-1R prevents rapamycin-induced AKT activation and sensitizes tumors to mTOR inhibition support combination mTOR/IGF-1R inhibition [22]. Unfortunately, anti-IGF-1R monotherapy has had limited activity in NET patients [23] and the combination of the anti-IGF-1R antibody cixutumumab and everolimus (plus octreotide) resulted in mainly stable disease and challenges with long-term tolerability [24].

A recent study has suggested that the PI3K/Akt/mTOR signaling crosstalks with the CXCR4-CXCL12-CXCR7 chemokine receptor axis. CXCR4, CXCL12, and CXCR7 are overexpressed in NETs and are associated with a higher tumor grade and advanced tumor stage [25]. The addition of a CXCR4 antagonist (AMD3100 (plerixafor)) to everolimus (RAD001) potentiated cell growth inhibition in a bronchial-NET cell line (NCI-H727) and the BON cell line, suggesting that crosstalk with the CXCR4-CXCL12-CXCR7 chemokine receptor axis may be another mechanism for everolimus resistance.

In addition to the combinations described above, studies including NETs patients have tested everolimus with HSP90 inhibition (SNX-5422) [26], with anti-angiogenic therapy (vorolanib) [27], and with the immunomodulatory agent lenalidomide [28]. In the HSP90 study, among 14 NET patients, there were two partial responses (14%) and eight instances of stable disease (57%). Vorolanib, an anti-VEGFR/PDGFR/CSF1R tyrosine kinase inhibitor, was combined with everolimus in solid tumor patients including NETs, and demonstrated dose-limiting toxicities in three patients (fatigue, hypophosphatemia, and mucositis). In addition, among fifteen evaluable patients, there were three with partial response (one of whom had NET) and eight with stable disease (six of whom had NET). Everolimus has been combined with the anti-VEGFR multikinase inhibitor sorafenib, demonstrating activity but also toxicity concerns [29]. Another study of everolimus plus an anti-VEGFR TKI (lenvatinib) is underway for NETs patients (NCT03950609). In the lenalidomide plus everolimus study in patients with advanced solid tumors, full-dose everolimus could be administered. Overall, there were partial responses in 13.8% and stable disease in 55.8%, although there was no specific mention of the neuroendocrine patients. Metformin, which decreases insulin and IGF-1 levels and causes AMPK activation, which inhibits the TSC1-2/mTOR complex, is being tested along with octreotide and everolimus [30], though results have not been reported.

### 3.2. Molecular Mechanisms of Resistance to Multi-Kinase Inhibitors

**Figure 2:**
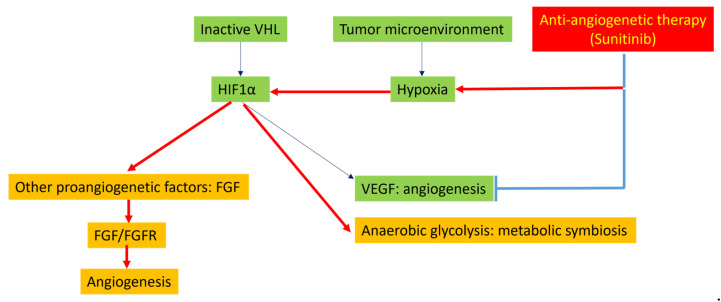
Potential mechanisms underlying resistance to sunitinib. Inhibition of vascular proliferation by sunitinib leads to hypoxia. Hypoxia leads to activation of HIF1α. HIF1α, in turn, promotes angiogenesis through activation of other proangiogenetic factors such as FGF. HIF1α also promotes anaerobic glycolysis, featured by metabolic symbiosis.

PanNETs are highly vascularized tumors with an overexpression of vascular endothelial growth factor (VEGF), platelet-derived growth factor (PDGF), and their receptors. Sunitinib, an inhibitor for several tyrosine kinases, including VEGF receptors, PDGF receptors, Fms-related receptor tyrosine kinase 3 (FLT-3), stem cell factor receptor, rearranged during transfection (RET), colony stimulating factor 1 receptor (CSF1R), and glial-cell-line-derived neurotrophic factor (GDNF), was FDA-approved for the management of advanced, progressive, and metastatic PanNETs [31], with a PFS of 11.4 months compared with 5.5 months for placebo. The hypoxia-mediated induction of other proangiogenic factors has been proposed to be the key mechanism for sunitinib resistance in PanNETs (Figure 2).

HIF-1α is thought to be the key driver of angiogenesis in PanNET [32]. HIF-1α can be activated by two mechanisms in PanNETs [7,32]. First, HIF-1α is regulated by the von Hippel-Lindau tumor suppressor (VHL); the genetic or epigenetic inactivation of VHL can lead to an accumulation of HIF-1α. Second, tumor hypoxia can also activate HIF-1α. Activated HIF-1α stimulates the transcription of hypoxia-related genes, such as VEGF and genes involved in anaerobic glycolysis (e.g., carbonic anhydrases IX). Immunohistochemistry has demonstrated an overexpression of HIF-1α, VEGF-A, and carbonic anhydrases IX in most GEP-NETs, suggesting their contribution in PanNET angiogenesis [33]. In addition to hypoxia-related genes, other pathways can be activated by HIF-1α, including several proangiogenic factors, such as fibroblast growth factors (FGFs), ephrins, and angiopoietins. The FGF/FGF receptor (FGFR) axis was demonstrated to be one of the key drivers of the VEGF-independent revascularization of PanNETs [34,35]. In the RIP-Tag2 mouse model of PanNET, a model also used for anti-angiogenesis research, brivanib, a dual FGF/VEGF inhibitor, produced enduring tumor stasis and angiogenic blockade following the failure of DC101 (an anti-VEGFR2 monoclonal antibody) or sorafenib [35]. Therefore, the hypoxic state following anti-angiogenic therapy may stimulate HIF-1α, which, in turn, activates VEGF-independent angiogenetic pathways, including FGF/FGFR, promoting tumor revascularization and, eventually, tumor progression. Therefore, the use of other multikinase inhibitors with VEGFR-tyrosine kinase inhibitor (TKI) inhibitory activity may have promise, despite progression on one such therapy. In the GETNET1509 clinical trial, lenvatinib, an inhibitor of VEGFR1-3, FGFR1-4, PDGFRα/β, RET, and c-KIT, demonstrated efficacy in PanNET patients with disease progression following treatment with other targeted agents [36].

In addition, metabolic symbiosis may be involved in adaptive resistance to anti-angiogenic therapy. In the RIP1 Tag2 mouse PanNETs model, acute hypoxia caused by angiogenesis inhibitors elicited metabolic compartmentalization, where hypoxia tumor cells imported and metabolized glucose and secreted lactate, whereas normoxic vessel-proximal tumors imported and metabolized lactate, illustrating the remarkable plasticity of tumor cells in response to treatment [37]. Metabolic symbiosis may also be dependent on the mTOR signaling pathway as mTOR signaling was upregulated and mTOR signaling inhibition disrupted the metabolic symbiosis.

Finally, sunitinib can induce lysosomal membrane permeabilization and, consequently, autophagy [38]. Lysosomal sequestration has been suggested to be a novel mechanism of sunitinib resistance. In colon and renal cancer cells, the intracellular sunitinib concentration was higher in resistant cells than in sensitive cells because of an increased lysosomal sequestration [38]. A recent study demonstrated that the accumulation of sunitinib in lysosomes in PanNETs induced autophagy and that chloroquine, an autophagy inhibitor, increased sunitinib efficacy in PanNET treatment [39]. Therefore, lysosomal sequestration and autophagy are potential mechanisms leading to PanNET resistance to sunitinib.

### 3.3. Epigenetic/Genetic Dysregulation and Therapy Resistance

Epigenetic modification and genetic alteration may contribute to the development of resistance to targeted therapies in small intestinal NET (SINET) and PanNET. SINETs have a low rate of mutations compared to other malignancies, suggesting that epigenetic dysregulation and copy number variation may be key mechanisms underlying tumor progression and metastasis [40]. An integrated genomic analysis of a large cohort of SINET liver metastases revealed significantly increased copy number variants (CNVs) in liver metastasis compared to primary tumors [41]. In addition, an analysis of 850,000 methylation sites demonstrated more epigenetic dysregulation in the metastases [42]. The accumulation of CNVs and epigenetic alterations may therefore be driving forces in resistance to targeted therapies in SINETs.

Although research on the role of epigenetic alteration in therapeutic resistance in SINET is limited, the important role of epigenetics has been investigated in PanNETs. Based on RNA sequencing and tumor DNA methylation profiling, PanNETs have recently been classified into (1) tumors with an α-cell signature (carrying *ATRX*, *DAXX*, and *MEN1* mutations with a high ARX and low PDX1 gene expression due to PDX1 promoter hyper-methylation) and (2) tumors with no *ATRX*, *DAXX*, and *MEN1* mutations [43]. PanNETs with an α-cell signature showed an increased expression of hepatocyte nuclear factor 1α (HNF1α) and its transcriptional target genes, and were associated with an adverse clinical outcome. Genetic sequencing studies on PanNETs have revealed a relatively stable genome but gene mutations affect several pathways, including (1) chromatin-remodeling genes (*DAXX*, *ARTX*, *MEN1*, and *SETD2*), DNA repair genes (*CHEK2, BRCA2*, and *MUTYH*), mTOR-related genes (*TSC2*, *PTEN*, and *PI3KCA*), and the oxygen-sensing modulator *VHL*. It has been suggested that primary and metastatic PanNETs share similar genetic alterations [44]. Multiple HDAC subtypes are significantly upregulated in high-grade (G3) PanNETs but not in G1 or G2 tumors [45]. The role of genetic mutations in therapy resistance has not been specifically investigated.

Tumor heterogeneity is common in GEP-NETs, which may be related to genetic heterogeneity and may contribute to drug resistance in some GEP-NETs. Tang LH et al. reported 31 well-differentiated PanNETs with a morphologically apparent high-grade component, and did not reveal additional mutations, including *TP53* mutations, in the high-grade component [46]. However, Martin, et al. analyzed a PanNET with focal high-grade progression and found an increased CNV and additional mutations in *PTEN* and *SMAD4* only in the high-grade component, suggesting that genetic heterogeneity is seen in rare PanNETs [47]. Consistent with this study, we have also observed that rare PanNETs with a focal high-grade component can have *TP53* mutations in addition to gene mutations commonly present in well-differentiated NETs.

**Figure 3:**
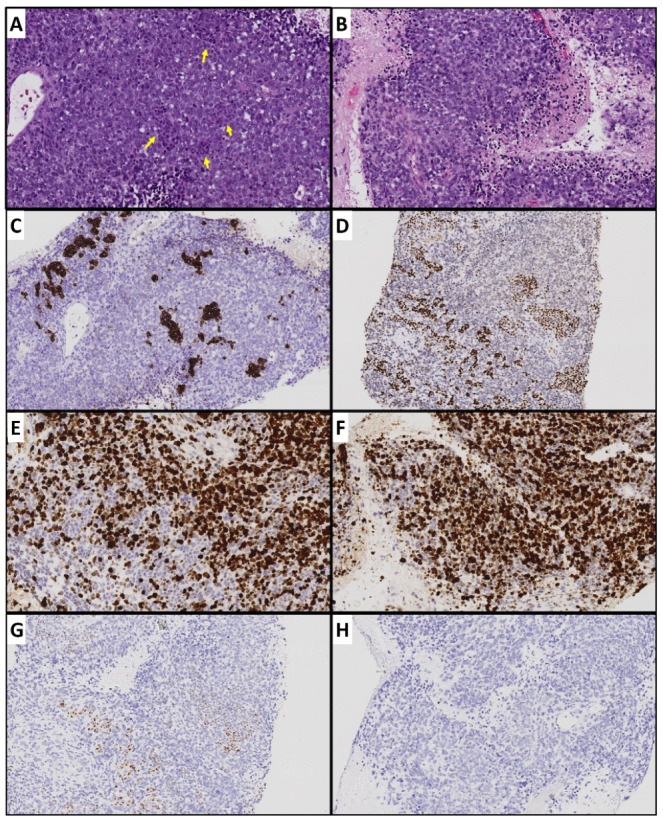
Metastatic de-differentiated carcinoma in a patient with well-differentiated pancreatic neuroendocrine tumor. (**A**) Representative tumor section containing foci of well-differentiated component (yellow arrows). (**B**) Poorly differentiated carcinoma with extensive necrosis and abundant apoptosis and mitoses. (**C**) Well-differentiated foci with synaptophysin expression but no expression in poorly differentiated component. (**D**) Well-differentiated foci with strong INSM-1 (another neuroendocrine marker) expression. (**E**,**F**) Relatively low Ki67 index in well-differentiated component and high Ki67 poorly differentiated component. (**G**) Normal p53 expression in well-differentiated component. (**H**) Complete loss of p53.

Although most primary and metastatic PanNETs share similar genetic alterations [44], additional genetic alterations may occasionally occur during metastasis/treatment. Recently we encountered a case of metastatic PanNET in which the primary tumor was WHO grade 2 by Ki67, but a subsequently biopsied liver lesion, while, morphologically, a well-differentiated NET with a strong expression of neuroendocrine markers had a much higher Ki67% (approximately 55%). A year after receiving everolimus, bevacizumab, and chemotherapy, the patient had progression in hepatic metastatic disease. A liver mass was biopsied again and showed poorly differentiated carcinoma intermixed with small foci of well-differentiated NETs (Figure 3). The poorly differentiated component showed extensive tumor necrosis, vesicular nuclei, and frequent mitoses. Immunohistochemistry showed that the poorly differentiated component was essentially negative for neuroendocrine markers with a loss of p53 expression. On the other hand, the small foci expressed neuroendocrine markers with a normal p53 staining pattern. The findings may represent “dedifferentiation”, likely due to an acquired *TP53* loss, which may contribute to therapy resistance. In addition, Wong, H.L. et al. identified a homozygous pathogenic *TP53* mutation, a gene fusion event involving *APC* and *MYCN* amplification in a resistant PanNETs [48]; however, the primary tumor was not tested for *TP53* alterations.

Other gene mutations may also contribute to drug resistance. Allen A et al. reported *BRAF* mutations in rare PanNETs [49]. The mutations included V600E and non-V600E mutations. A functional analysis revealed that these mutations detected in PanNETs were associated with MAPK pathway activation, which was abrogated by RAF and MEK inhibitors in vitro. *BRAF* mutations were identified in progressive metastatic lesions after multiline therapies, and, in one case, a *BRAF* mutation was not detected in one metastatic lesion, though it was detected in the others. These findings suggest that *BRAF* mutations may be responsible for drug resistance in rare PanNETs and that NGS on progressive lesions following therapy may be helpful for identifying novel therapeutic targets.

## 4. Resistance to SSTR-Targeted Therapy

Somatostatin receptors, including SSTR1, SSTR2, SSTR3, SSTR4, and SSTR5, are frequently expressed in GEP-NETs. Among them, the most commonly expressed is SSTR2, which has become an important target for therapy in NETs with either the somatostatin analogues, octreotide and lanreotide, or the radiolabeled SSTR2 binding analogue Lu177-DOTATATE. Long acting/depot forms of octreotide and lanreotide, typically administered as first-line therapy for advanced NETs and for managing carcinoid syndrome, activate the SSTR2 and SSTR5 receptors and control hormone secretion through the inhibition of voltage-dependent calcium channels, generation of cAMP by adenylyl cyclase, and inhibition of proliferation by activating pro-apoptotic pathways and phosphatases that downregulate PI3K/Akt and MAPK pathways [50]. When the ^177^Lu radiolabeled somatostatin analogue ^177^Lu-DOTATATE binds to SSTR2 on tumor cells, the complex is internalized, causing ionizing radiation, DNA damage, and, eventually, cell death [51].

In the PROMID study in patients with midgut NETs [52] fewer than 20% of patients in the octreotide LAR-treated group progressed in the first 6 months, whereas approximately 50% receiving placebo did; however, more than 70% treated with octreotide LAR had progressed on the long-term follow-up. Although many patients continue to receive SSAs beyond progression on the assumption that there is still activity and that higher doses can occasionally temporarily control disease [53], it is still likely that these data are suggestive of resistance. Recent studies and clinical trials have demonstrated that PRRT with a radiolabeled SSA (^177^Lu-DOTATATE) induces significant antitumor effects in patients with metastatic SSTR2 expressing GEP-NETs (reviewed in [54]), leading to the FDA’s approval of 177Lu-DOTATATE for the treatment of somatostatin receptor-positive gastroenteropancreatic neuroendocrine tumors in adults; however, approximately 15–30% of patients will have disease progression during therapy (primary resistance). In addition, a complete response is rare and progression occurs in the majority (acquired resistance).

### 4.1. Tumor Heterogeneity

**Figure 4:**
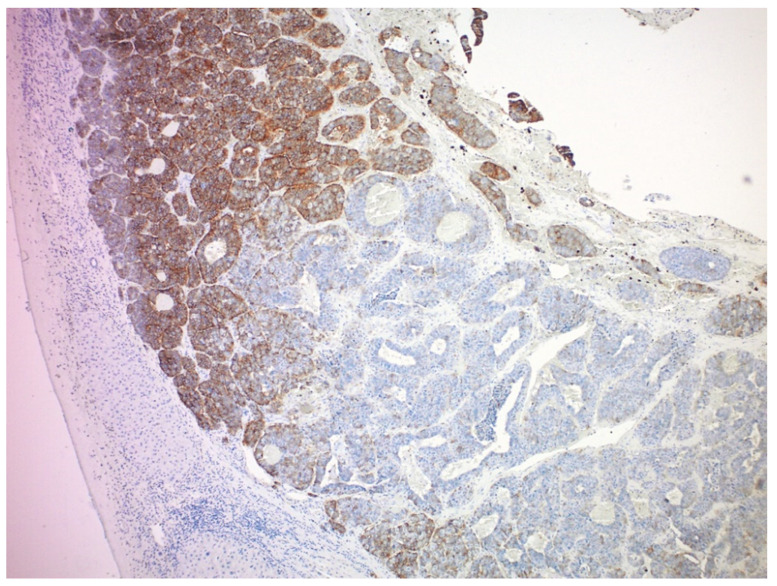
A representative section of liver metastasis from a small intestinal neuroendocrine tumor showing heterogeneous SSTR2 expression.

SSTR2 expression is associated with a longer progression-free survival in patients treated with SSAs [55]. Therefore, it is possible that a mechanism of resistance to SSAs could be the downregulation of SSTR expression, though it has not been reported thus far. Similarly, defects in downstream mediators of the pro-apoptotic anti-proliferative effects of SSTR2 agonism could potentially explain resistance, but this has not been reported previously. In patients with metastatic NETs treated with Lu177-DOTATATE, the extent of somatostatin receptor expression and tumor heterogeneity (as determined by the coefficient of variation, kurtosis, and skewness) is associated with the response and PFS [56]. Feijtel and colleagues analyzed PRRT-induced radiobiological responses in SSTR2-expressing cell lines and xenografted mice and found that heterogeneous SSTR2 expression levels within NETs caused differentially induced DNA damage levels and that inter and intra-tumor SSTR2 heterogeneity influenced the PRRT response [57]. Although SSTR2 is highly expressed in most GEP-NETs, its expression can be heterogeneous. Both inter-tumoral and intra-tumoral heterogeneous SSTR2 expression (Figure 4) can be seen in metastatic GEP-NETs. We analyzed SSTR2 expression in 156 liver metastases from 26 patients with at least two resected liver lesions [58]. We found that although most liver metastases had a moderate to strong SSTR2 expression, approximately 44% of the patients had at least one liver lesion showing only weak to no SSTR2 expression. In addition, the intratumoral heterogeneous expression of SSTRs is frequent in both metastatic and primary tumors, especially in large liver tumors. As a β-emitter with an average penetration range of 0.67 mm, ^177^Lu may be unable to kill neighboring SSTR-negative cells in large tumors with heterogeneous expression [59]. The relevance of this issue is unclear though, and there may be less damage to normal nearby tissue due to the shorter penetration range. Further, in the NETTER 1 study, there was no difference in outcome for those with Krenning score 4 uptake compared with those with a lower score [60], although the resolution of the somatostatin receptor imaging may be inadequate for detecting the heterogeneous SSTR expression. One future solution to the heterogeneity of SSTR2 expression may be using a combination of Y90 radioembolization for larger tumors and ^177^Lu-DOTATATE for smaller tumors.

The radiation emitted from ^177^Lu principally induces single-strand breaks [61], but it may also lead to cell death through double-strand DNA breaks [62], the latter being dependent on cell cycling. Along with others, we have observed considerable intra-tumoral Ki67 proliferation index heterogeneity and differences between primary and metastatic sites [63], which could lead to a differential sensitivity to radiotherapy emitted by ^177^Lu-DOTATATE. However, a recent study suggested that an intra-tumor heterogeneity of DNA damage and apoptosis following PRRT was not attributed to proliferation in mouse models [57]. Human studies also suggest that higher-grade (higher Ki67) tumors had less of a clinical benefit from PRRT [64]. Therefore, more data will be needed to determine whether alterations in Ki67 may explain the development of resistance to ^177^Lu-DOTATATE.

### 4.2. Tumor Hypoxia Related to Peritoneal Metastasis and Mesenteric Tumor Mass

Peritoneal metastases, present in 20% of SINETs patients [65], and mesenteric masses found in >50% [66], may provide a specific resistance challenge to the activity of 177Lu-DOTATATE. Almost 40% of patients with diffuse GEP-NET peritoneal metastases showed peritoneal progression during PRRT with ^177^Lu-DOTATATE [67]. Peritoneal metastasis is characterized by hypoxia and the formation of a vascularized connective tissue stroma mediated by VEGF [68]. The presence of hypoxic regions within tumors may be a main basis for radiotherapeutic failure in some tumors [69]. It is believed that oxygen is required to induce damage to DNA during radiotherapy and that hypoxia leads to radioresistance. In addition, hypoxia results in altered genetic pathways that promote cell survival despite an adverse tumor microenvironment. Hypoxia can stimulate HIF-1α, and HIF-1α, in turn, enhances the expression of genes involved in glycolysis (GLUT1) and angiogenesis (VEGF), promoting tumor growth. Further hypoxia can induce a selection of clones that are resistant to apoptosis.

**Figure 5:**
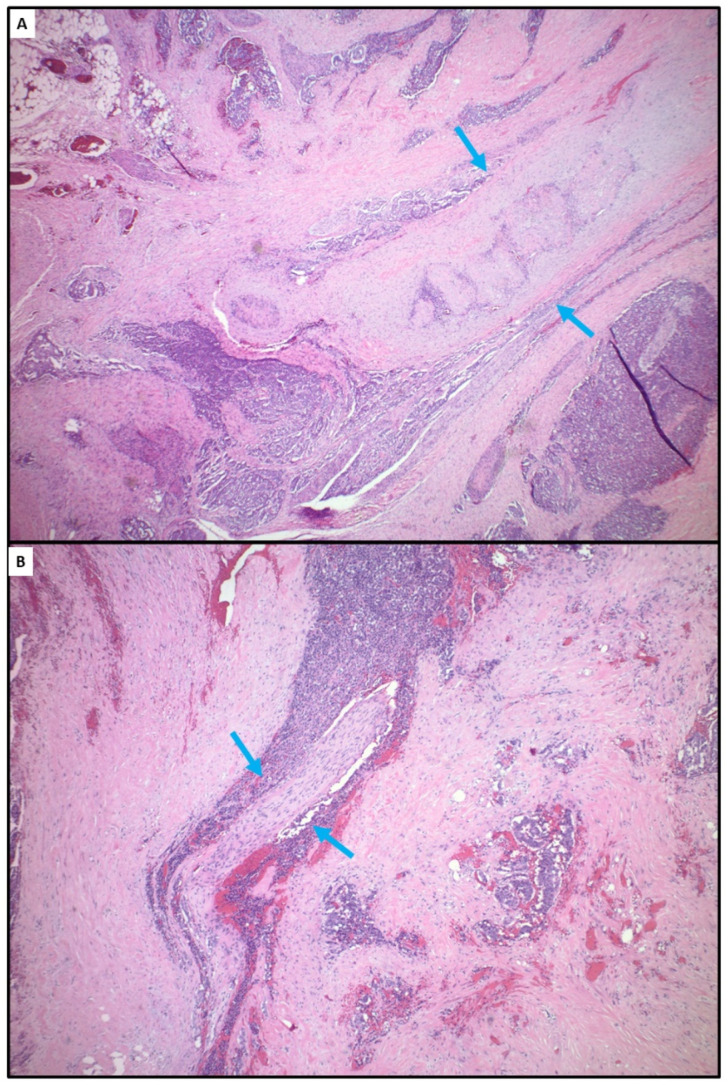
Representative sections of a mesenteric mass from a patient with small intestinal neuroendocrine tumor showing extensive fibrosis. (**A**) Entrapped large mesenteric artery with elastosis (blue arrows). (**B**) Entrapped nerve bundle (blue arrows).

Mesenteric masses seen in patients with SINET are frequently associated with extensive fibrosis (Figure 5). The molecular basis of the mesenteric fibrosis has not been elucidated. Nevertheless, extensive fibrosis may lead to a locoregional hypoxic tumor microenvironment, which may affect radiosensitivity. In addition, ^177^Lu has a short range of radiation, and is effective in localizing cytotoxic radiation in relatively small areas. The paucity of vasculatures due to dense fibrosis may prevent the peptide–chelator–radionuclide complex from accessing areas with extensive fibrosis, which may contribute to ^177^Lu DOTATATE resistance. PRRT treatment was shown to result in an objective size reduction in mesenteric masses in only 3.8% of the patients [70].

## 5. Future Directions

Multikinase inhibitors with novel combinations of activities may be effective after or in place of existing TKIs. For example, surufatinib, a small-molecule tyrosine kinase inhibitor, has activity against the VEGFR 1-3, FGFR 1, and CSF-1 receptor. This combination of targets may result in the inhibition of angiogenesis, tumor-immune evasion, and tumor resistance [71]. In preclinical models, epigenetic modulation with HDAC inhibitors may redifferentiate neuroendocrine tumors and resensitize them to prior therapies [72]. In a pilot study, the HDAC inhibitor vorinostat was associated with a greater DOTATATE uptake (by PET imaging) [73]. Other strategies for targeting SSTR not encumbered with resistance mechanisms against radioactivity may be options. Preclinical work identified an antibody drug conjugate against SSTR2 [74]. Identifying other surface molecules as targets for tumor directed therapies might also be necessary. In this same study, NETs found to have low or no expression of SSTR2 expressed CEACAM1, a different potential target.
